# Metabolic Profiling and Physiological Analysis of a Novel Rice Introgression Line with Broad Leaf Size

**DOI:** 10.1371/journal.pone.0145646

**Published:** 2015-12-29

**Authors:** Xiuqin Zhao, Guilian Zhang, Yun Wang, Fan Zhang, Wensheng Wang, Wenhao Zhang, Binying Fu, Jianlong Xu, Zhikang Li

**Affiliations:** 1 Institute of Crop Sciences/National Key Facility for Crop Gene Resources and Genetic Improvement, Chinese Academy of Agricultural Sciences, Beijing 100081, China; 2 Shanghai Landscape Gardening Research Institute, Shanghai 200232, China; 3 Rice Institute of Shenyang Agricultural University/Key Laboratory of Crop Physiology, Ecology, Genetics and Breeding, Ministry of Agriculture, Shenyang 110866, China; 4 China University of Mining and Technology, Beijing 100083, China; 5 Shenzhen Institute for Innovative Breeding, Chinese Academy of Agricultural Sciences, Shenzhen 518120, China; 6 Agricultural Genomics Institute, Chinese Academy of Agricultural Sciences, Shenzhen 518120, China; Institute of Genetics and Developmental Biology, Chinese Academy of Sciences, CHINA

## Abstract

A rice introgression line, NIL-*SS1*, and its recurrent parent, Teqing, were used to investigate the influence of the introgression segment on plant growth. The current research showed NIL-*SS1* had an increased flag leaf width, total leaf area, spikelet number per panicle and grain yield, but a decreased photosynthetic rate. The metabolite differences in NIL-*SS1* and Teqing at different developmental stages were assessed using gas chromatography—mass spectrometry technology. Significant metabolite differences were observed across the different stages. NIL-*SS1* increased the plant leaf nitrogen content, and the greatest differences between NIL-*SS1* and Teqing occurred at the booting stage. Compared to Teqing, the metabolic phenotype of NIL-*SS1* at the booting stage has closer association with those at the flowering stage. The introgression segment induced more active competition for sugars and organic acids (OAs) from leaves to the growing young spikes, which resulted in more spikelet number per plant (SNP). The results indicated the introgression segment could improve rice grain yield by increasing the SNP and total leaf area per plant, which resulted from the higher plant nitrogen content across growth stages and stronger competition for sugars and OAs of young spikes at the booting stage.

## Introduction

Rice (*Oryza sativa* L.) is a staple food crop worldwide, especially in developing countries. It is predicted that a 26% increase in rice production will be required to feed the rising population by 2035, without a corresponding increase in the amount of agricultural land [1, 2]. Therefore, developing high yield varieties is becoming one of the most important goals of rice breeding worldwide [3].

Green plants, unlike animals, produce their own organic compounds, and their growth solely depends on their own photosynthetic and metabolic capacity [4]. The basic premise of high yield in plants is to improve leaf photosynthetic efficiency and coordinate the source—sink relationship [5]. As a dominant primary source of assimilates, the leaves’ photosynthetic capacity plays an important role in determining crop yield [6]. The flag leaf is the main source organ [6–8], and its capacity to photosynthesis and produce photoassimilates is determined by leaf area and nitrogen content per unit leaf area [9,10]. The sink organs are net importers of assimilates: the sinks are dynamic and can be growing leaves, spikelets or filling grains. An increase in the grain number and grain size (sink capacity) is always accompanied by a higher dry-matter accumulation capacity of crops [11,12].

Recently, many yield component genes have been identified and many have been cloned using map-based cloning strategy, for example, grain size related genes, *gw2* [13], *qSW5* [14] and *GS3* [15]; grain filling gene, *GIFI* [16]; panicle number genes, *OsSPL14* [17,18] and *DEP1* [19]; spikelet number genes, *Gna1*[20] and *qSSP7* [21]; and yield pleiotropic genes, *Ghd7* [22] and *Ghd8* [23]. Few of them could be used to markedly improve the yield by molecular breeding [6, 24], which illustrated the complexity of yield formation [6] and that better yield could be achieved by a successful regulation of source—sink relationships in the production and utilization of photosynthates within a plant. Neither source nor sink manipulation alone can improve crop yield indefinitely [12]. Recently, the *NARROW LEAF1 (NAL1)* gene was found to be related to higher grain yield in rice, and physiological analysis showed that the *NAL1* controlled the source and sink simultaneously, which has great potential in yield breeding [6, 24,25].

To the best of our knowledge, even though there are many reports on genes related to rice yield, there have been few on functional metabolites related to yield. Metabolite profiling is being increasingly used to investigate metabolic regulation of the systemic response to the environment or to decipher gene function in plants [26–30] and it is believed that integrating the results from metabolic profiling and morphology would be a powerful strategy for crop improvement [30–32].

As part of our rice breeding program comprising introgressing diverse germplasm into elite varieties [33], we developed a near-isogenic line (NIL), NIL-*SS1*, the introgression segment from Lemont into the Teqing background harbored with sink—source related gene, *SS1* [25]. NIL-*SS1* has broader leaves, higher spikelet number per panicle and higher grain yield than the recurrent parent, Teqing. After fine-mapping and map-based cloning [25], *SS1* was identified as allelic to *NAL1*[34], *SPIKE* [24] and *GPS* [6], which were reported to have effects on leaf size, the number of vascular bundles, spikelet number and photosynthesis, resulting in increased grain yield. Understanding the relative physio—biochemical mechanisms for higher yield regulated by the introgression segment harbored with *SS1* would be beneficial to rice breeding. In the present study, the specific performance of NIL-*SS1* was characterized at the phenotypic level of the source and sink. Moreover, the time-course of metabolites change patterns in leaves of NIL-*SS1* were also investigated. The results will shed light on the photo—physiological function of *SS1* and the metabolic basis for its high yield performance.

## Materials and Methods

### Plant materials and growth conditions

The near isogenic line, NIL-*SS1*, and recurrent parent, Teqing, were used as materials in this study. Teqing is a high-yielding semidwarf *indica* rice variety from China; and NIL-*SS1* was developed as reported [25] which containing the Lemont (a commercial semidwarf *japonica* rice variety from Southern US) allele at *SS1* in a small region of 50.3 kb flanked by markers RM3534 and FL98 on chromosome 4.

Two experiments were conducted in two consecutive years. In 2009, photosynthetic traits were investigated in the screening house of the Chinese Academy of Agricultural Sciences (CAAS), Beijing. Rice plants with four leaves were transplanted into three-row plots, with 12 plants in each row (36 plants per plot) at a spacing of 25 × 15 cm for three replications. The plants were managed according to standard experimental procedures; In 2010, the relative traits were investigated at two sites. The plants were cultivated in the same way as those in 2009 under fully irrigated condition in the field experimental farms of CAAS and Guangdong province, China, respectively. The relative physiological traits and metabolites were systematically evaluated at the tillering, booting, flowering and grain filling stages, respectively, in CAAS, and yield were evaluated again in Guangdong site which is the place of origin for variety, Teqing.

### Physiological trait evaluation

#### Photosynthetic rate

The photosynthetic trait analyses were conducted in two consecutive years. In 2009, the traits were investigated at the flowering stage only, and in 2010, the traits were investigated at four growth stages, *i*.*e*., tillering, booting, flowering and grain filling stages. The leaf photosynthetic net rate (*P*_*N*_) was measured in the topmost fully expanded leaf using a portable photosynthesis system (Li-Cor6400, Li-Cor Inc., Lincoln, NE, USA). The measurements were made from 9:00 to 11:20 on a sunny day. The photosynthetically active radiation was set to 1,500 mol m^−2^ s^−1^ and was supplied mixed red/blue LEDs.

#### Flag leaf width (FLW) and total leaf area per plant (TLA)

The two leaf traits were investigated across the four stages. The FLW, the topmost fully expanded leaves of the main stems of five different plants in each plot were sampled and measured for FLW; the TLA was calculated as the total area of all leaves per plant, three plants of each genotype in each plot were investigated.

#### Yield and yield components

Grain yield and spikelet number per panicle (SNP) were investigated after harvest in 2010.

### Metabolite extraction and identification

The topmost leaves of five plants were sampled for both genotypes at tillering, booting, flowering and grain filling stages, respectively; three replicates were performed for each sample. All samples were flash-frozen in liquid nitrogen, stored at −70°C until metabolite extraction. Metabolite extraction was carried out as described by Bowne et al. [35] and Zhao [36]. The extracted samples were then derivatized and analyzed by gas chromatography-mass spectrometry (GC-MS). Chromatograms and mass spectra were processed using the find algorithm implemented in GC-MS Postrun Analysis software (Shimadzu). Specific mass spectral fragments were detected in defined retention time windows using the mass spectral libraries of NIST08, NIST08S and Wiley 9 and the public domain mass spectra library of the Max Planck Institute in Germany (http://csbdb.mpimp-golm.mpg.de/csbdb/). A mixture of the leaves of both genotypes at four stages were extracted in bulk and used as a reference. The reference samples were run once every ten samples.

### Data analysis

For subsequent statistical analyses of the metabolites, the relative signal intensities for the detected metabolites were normalized to the mean intensity of all reference samples. Analyses of variance (ANOVA) (SAS Institute Inc. 1996) was performed to determine the significances of the physiological traits differences and the metabolites differences between genotypes (G), development stages (S) and the interaction between genotype and development stages (G*S). Differentially altered traits or metabolites were defined as those showing significant concentration increases or decreases relative to the other genotype at *p*≤0.05. Moreover, the normalized metabolites were further analyzed: hierarchical clustering was performed using the *hclust* function in the software R (publicly available at www.r-project.org), which uses Euclidean distance as a measure of similarity between data points. The *heatmap* function in *R* was used to visualize the clustering results. Principal component analysis (PCA) was conducted using the SPSS software. Mapman software was used to display the comprehensive changes in metabolites participating in different pathways between NIL-*SS1* and Teqing.

## Results

### Comparison of morpho-physiological traits and grain yield between NIL-*SS1* and Teqing

The physiological analysis showed, in 2009, the leaf photosynthetic rate (*P*_*N*_) of NIL-*SS1* decreased by 18.4% compared with that of Teqing at the flowering stage (Table 1). In 2010, the *P*_*N*_ was investigated at four growth stages. NIL-*SS1* had consistently lower *P*_*N*_ values than Teqing at the tillering, booting and flowering stages, but a higher value at the grain filling stage. For example, the *P*_*N*_ decreased by 12.4%, 11.9% and 11.0% in NIL-*SS1* at the former three stages, respectively, while it increased by 28.6% at grain filling stage than that in Teqing.

**Table 1:** The photosynthetic rate (*P*_*N*,_ μmol•m^−2^s^−1^), flag leaf width (FLW, cm), total leaf area (TLA, cm^2^• plant^−1^), in the leaves of Teqing (TQ) and NIL-*SS1* (*SS1*) at different growth stages and the spikelet number per panicle (SNP), grain yield (g•plant^−1^) in 2009 and/or 2010.

	Location	G	Stage	*P*_*N*_	FLW	TLA	SNP	Yield
2010	Beijing	TQ	Tillering	26.6 ±0.70	0.81 ±0.011	1592.6 ±85.1		
		*SS1*		23.3 ±1.17*	0.89 ±0.012*	1888.7 ±76.5*		
		TQ	Booting	15.7 ±0.50	1.09 ±0.013			
		*SS1*		13.8 ±0.11*	1.33 ±0.011*			
		TQ	Flowering	22.1 ±0.44	1.42 ±0.003	2906.9 ±98.2		
		*SS1*		19.7 ±1.05*	1.75 ±0.121*	3471.4 ±112.9*		
		TQ	Grain Filling	10.2±0.71	1.44 ±0.023	2062.5 ±61.4	238.2 ±10.5	34.2 ±0.30
		*SS1*		13.1 ±1.46 *	1.77 ±0.012*	2139.2 ±74.5	296.2 ±7.41*	35.5 ±0.22
	Guangdong	TQ	Grain Filling		1.51 ±0.031		259.3 ±12.3	37.1 ±0.55
		*SS1*			1.79 ±0.029*		305.6±11.6*	40.0 ±0.68*
2009	Beijing	TQ	Flowering	18.7±1.23				
		*SS1*		15.2 ±1.67*				

Note: The values were different significantly (*p* < 0.05) between NIL-*SS1* and Teqing were indicated with *

Morphological investigation showed that compared with Teqing, the NIL-*SS1* had higher levels in flag leaf width (FLW) and total leaf area (TLA) across all growth stages (Table 1). For example, the FLW increased significantly by 9.9%, 22.0%, 23.2% and 22.9% at four stages, respectively. The TLA increased by 15.7%, 16.3% and 3.6% at the tillering, flowering and grain filling stages, respectively. The lower TLA change value at the filling stage probably reflected the death of more old leaves. The grain yield and yield component were investigated. The results showed that compared to Teqing, the spikelet number per panicle (SNP) of NIL-*SS1* increased by 24.4% and 17.8%, respectively, and the yield increased by 3.8% and 7.88%, respectively, in Beijing and Guangdong in 2010. (Table 1).

### Overview of the changes in metabolites across the growth stages

The metabolite profiles in the leaves of both genotypes were investigated at four growth stages, and 79 metabolites were identified (S1 Table). These metabolites included 10 amino acids (AAs), 31 organic acids (OAs), 23 sugars and 15 other small molecular components (SMCs).

ANOVA results indicated the developmental stage (S) caused the most significant changes in metabolites (S1 Table), *i*.*e*., 62 measured metabolites were significantly influenced by the developmental stage, which, explained an average of 66% of the total phenotypic variation. A number of 14 metabolites showed significant genotype differences (G) between Teqing and NIL-*SS1*, which, explained an average of only 9.1% of the total phenotypic variation. Genotype by development stage interaction was significant for 24 metabolites and explained an average of 32.9% of the total phenotypic variation of the metabolites.

**Fig 1:**
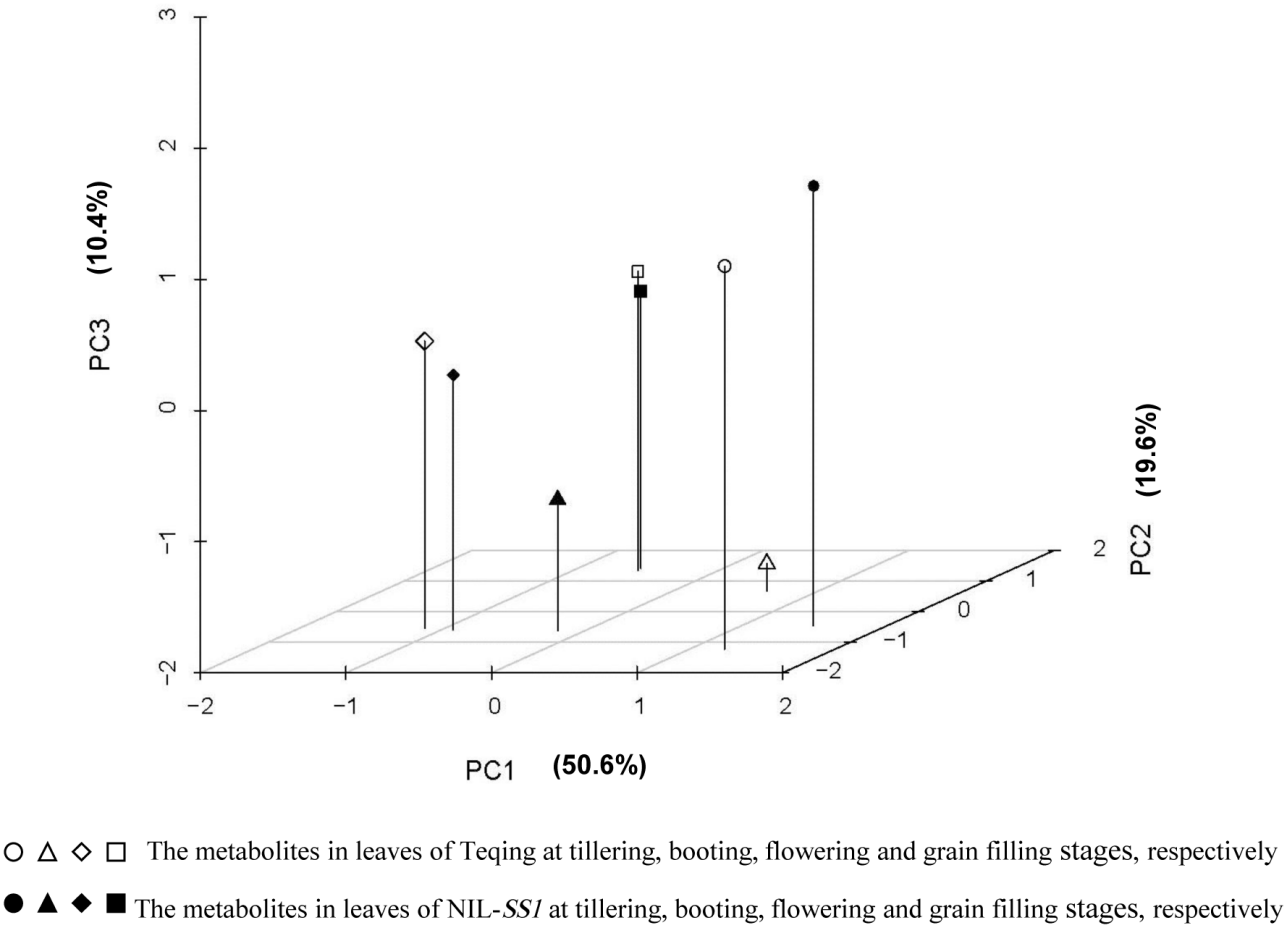
Principal component analysis (PCA) for metabolites in leaves of NIL-*SS1* and Teqing at the tillering, booting, flowering and grain filling stages.

To obtain an overall picture of the metabolite profiles of the two rice genotypes in response to developmental stage, the measured metabolite data were subjected to principal component analysis (PCA). As shown in Fig 1, the rice samples at different stages were separated clearly by PC1, which accounted for 50.6% of the total detected metabolite variance. The metabolic phenotypes of tillering stage were located at the X positive axis, which was significantly grouped out from the samples at other three stages. The results indicated potential differences in metabolic phenotypes between vegetative and reproductive stages. Loading analysis showed more OAs were significantly accumulated in leaves at the vegetative stage, while more sugars and AAs were accumulated at the reproductive stage (S2 Table). PC2 accounted for 19.6% of the variance, which clearly separated the two genotypes from each other at each stage, except for that at the grain filling stage, when the two genotypes were clustered together. Moreover, the opposite metabolic responses to growth stage between genotypes were observed (Fig 1). For example, along the Y-axis, the Teqing samples were distributed above the NIL-*SS1* at the booting stage, while distribution pattern was the opposite at the other three stages. Loading analysis showed that compared with those of Teqing, the levels of four OAs (cinnamic acid-4-hydroxy, hydroxypropanoic acid, azelaic acid, butyric acid-4-hydroxy and 2-monopalmitoylglycerol) were significantly decreased, while three AAs (glutamic acid, threonine and serine) were increased significantly in NIL-*SS1* at the booting stage (S2 Table). PC3 accounted for 10.4% of the variance, which clearly separated the booting stage samples from the others. The result indicated a specific metabolites pattern in leaves when the panicle was forming. Loading analysis indicated that two sugars (sucrose and melezitose), three OAs (threonic acid, malic acid and quinic acid and 5-p-coumaroyl) and one AA (phenylalanine) decreased significantly while aconitic acid, threonine and adenosine increased obviously in the leaves of rice at the booting stage (Fig 1; S2 Table).

**Fig 2:**
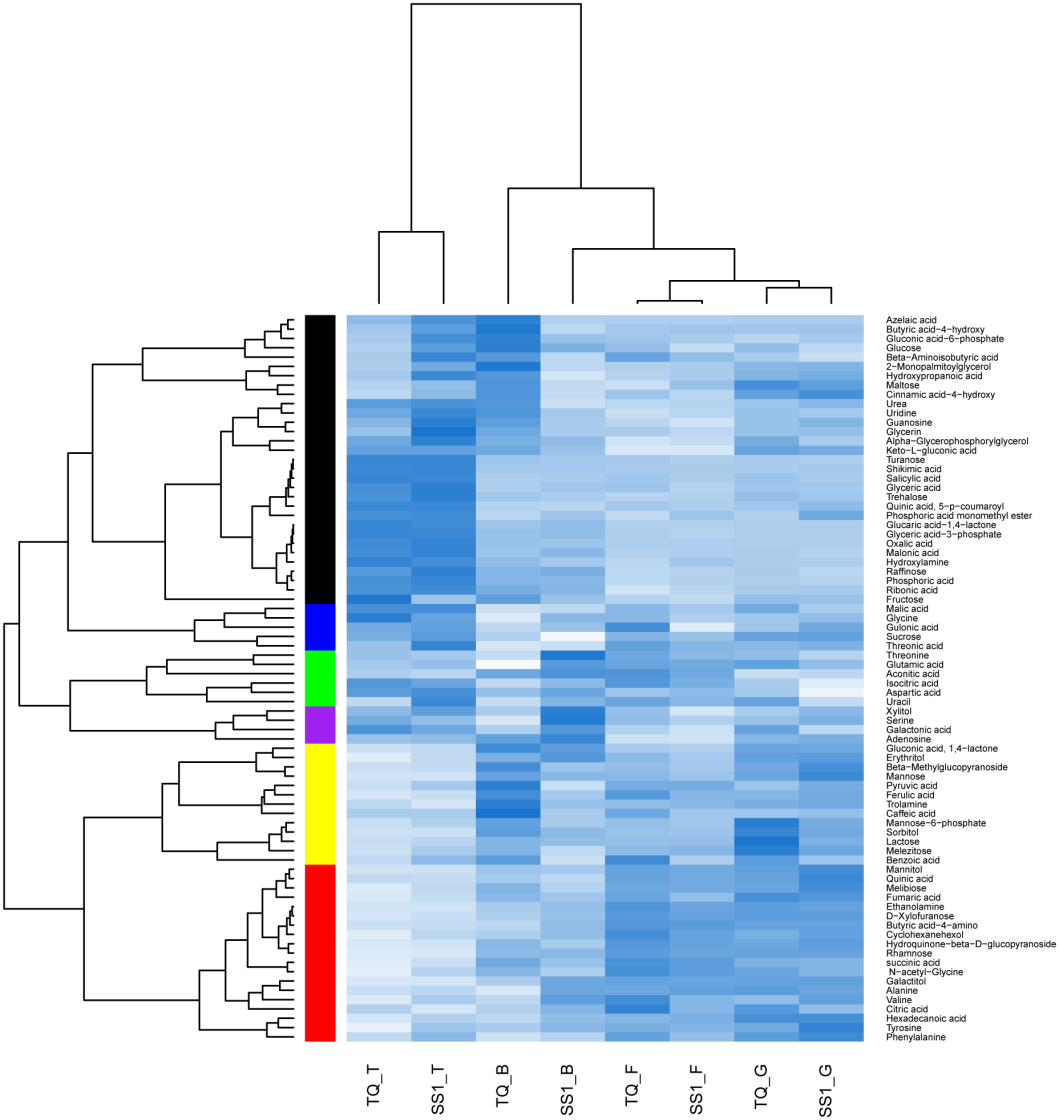
Hierarchical clustering analysis of the metabolite levels in leaves of Teqing (TQ) and NIL-*SS1* (*SS1*) at tillering (T), booting(B), flowering (F) and grain filling (G) stages using the R software.

Meanwhile, hierarchical cluster analysis was conducted to provide a global view of metabolite changes in Teqing and NIL-*SS1* at different developmental stages (Fig 2). The results confirmed that the developmental stage was the main determinant factor of metabolic phenotype. The metabolic phenotypes of the two genotypes at each developmental stage were very similar, except at the booting stage, when the genotypes were separated distinctly by the stage. The most significant genotype difference occurred at the booting stage. Compared with that of Teqing, the metabolic profile of the NIL-*SS1* was more similar to the samples at the latter stages.

### Comparison of metabolite levels in NIL-*SS1* and Teqing in leaves at different developmental stages

**Fig 3:**
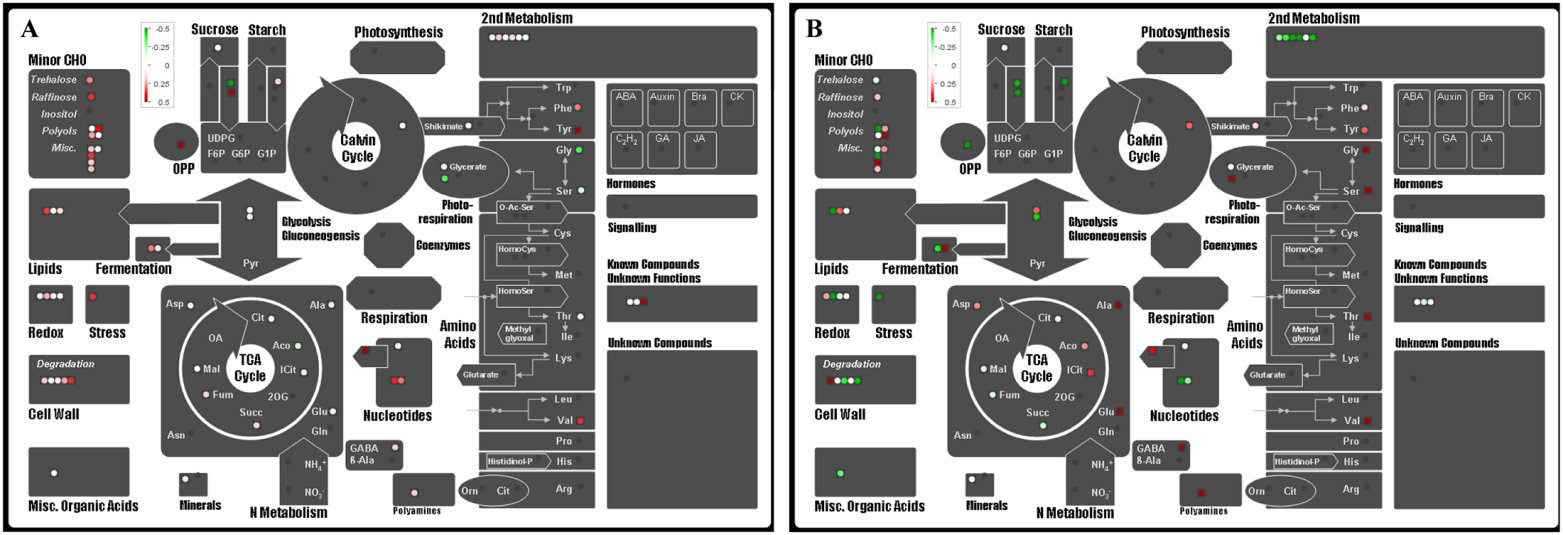
Display of changes of metabolites in NIL-*SS1* compared to that in Teqing. The samples were harvested at the tillering (A) and booting stage (B), respectively. A coloration represents 50% changes rate.

To get a comprehensive view for metabolites changes in different pathways, Mapman analysis was performed by using the data at the tillering and booting stages (Fig 3). The results showed that compared to that in Teqing, the levels of the minor sugars including trehalose, raffinose, metabolites involved in lipid metabolism, cell wall degradation, second metabolism and nucleotides metabolism were highly increased in NIL-*SS1* at the tillering stage; while, most of the changes observed at the tillering stage presented a negative change pattern at the booting stage, in addition, the metabolites related to starch and sucrose breakdown were observed decreased, while AAs and OAs participated in TCA were increased in NIL-*SS1*.

To determine the metabolites differences between the two genotypes, we further compared the levels of each metabolite in the NIL-*SS1* to those in the Teqing at the same growth stages using AVOVA (Table 2). Once the level of a metabolite showed a significant change (p ≤ 0.05 based on ANOVA) relative to Teqing, it was defined as differentially expressed one.

**Table 2:** Comparison of metabolite levels between leaves of NIL-*SS1* (*SS1*) and Teqing (TQ) at four different growth stages.

		Tillering		Booting		Flowering		Filling	
		TQ	*SS1*	TQ	*SS1*	TQ	*SS1*	TQ	*SS1*
**Amino acids**	Alanine	38.1	41.2	**33.8**	61.8*	61.0	63.8	66.6	62.1
	Aspartic acid	127.8	136.2	99.7	120.5	95.9	104.3	92.5	60.1
	Glutamic acid	97.4	104.2	**57.7**	135.6*	125.3	122.1	129.6	106.8
	Glycine	52.4	41.6	19.2	34.4	**34.7**	27.9*	30.0	33.0
	N-acetyl-Glycine	32.5	38.2	43.8	39.4	51.8	48.9	47.3	44.5
	Phenylalanine	53.4	65.4	50.9	56.4	73.2	62.5	75.3	80.6
	Serine	109.5	98.0	**70.6**	141.9*	98.3	85.0	95.2	106.5
	Tyrosine	**15.4**	31.7*	29.3	36.6	39.9	37.7	**41.2**	55.1*
	Valine	**75.0**	96.9*	88.1	127.2	**138.3**	110.5*	106.4	125.5
	Threonine	106.1	99.4	84.9	168.1	128.1	113.7	109.6	92.5
	**Average**	**70.7**	**75.3**	**57.8**	**92.2**	**84.7**	**77.6**	**79.4**	**76.7**
**Organic acids**	Aconitic acid	23.9	21.4	35.2	42.2	41.5	35.6	18.7	20.6
	Benzoic acid	34.6	39.0	**43.3**	33.7*	45.6	36.4	43.3	37.7
	Butyric acid-4-amino	29.3	32.9	35.8	55.3	80.5	78.4	73.5	72.3
	Butyric acid-4-hydroxy	**43.3**	70.8*	**97.9**	31.4*	41.0	44.2	42.7	45.3
	Caffeic acid	39.0	39.7	**104.3**	39.2*	**65.2**	42.4*	42.9	48.5
	Cinnamic acid-4-hydroxy	38.7	41.7	45.9	38.3	40.7	39.1	44.7	46.4
	Citric acid	42.5	40.5	42.3	44.9	49.1	44.7	47.3	44.0
	Quinic acid	69.9	67.0	102.6	74.2	175.9	160.7	173.3	222.5
	Ferulic acid	26.3	29.3	**70.3**	39.9*	63.7	49.8	50.1	55.7
	Fumaric acid	25.5	28.7	39.0	34.8	44.7	37.8	50.5	48.5
	Galactonic acid	43.8	41.0	35.8	43.1	35.4	33.2	41.5	34.4
	Gluconic acid-6-phosphate	49.7	81.7	88.3	54.8	51.8	49.6	44.1	50.2
	Glyceric acid	37.7	40.7	25.0	26.6	27.0	24.3	26.8	26.0
	Glyceric acid-3-phosphate	67.2	66.0	18.9	23.5	10.6	6.8	10.2	9.2
	Gulonic acid	40.3	41.2	34.9	37.3	43.5	32.5	36.9	40.4
	Hexadecanoic acid	24.4	29.1	**27.2**	33.2*	34.4	36.1	42.9	42.7
	Hydroxypropanoic acid	51.8	63.6	61.2	46.8	50.2	51.5	56.1	57.3
	Isocitric acid	60.3	54.9	37.0	47.7	60.5	51.9	41.7	30.2
	Keto-L-gluconic acid	32.1	32.1	31.7	30.1	27.7	27.7	31.9	31.3
	Malic acid	37.7	37.9	21.9	23.7	30.0	26.7	32.4	26.3
	Malonic acid	48.3	53.1	**23.9**	29.6*	18.6	19.3	**20.6**	17.5*
	Oxalic acid	37.9	40.5	9.2	7.4	1.9	2.4	2.9	2.2
	Phosphoric acid	41.0	44.1	30.4	30.2	21.3	23.3	24.1	22.5
	Pyruvic acid	**48.8**	52.9*	**66.4**	48.9*	58.2	58.1	**53.6**	58.3*
	Salicylic acid	35.4	34.9	20.2	20.8	22.0	20.4	22.6	20.9
	Shikimic acid	26.1	27.1	1.0	1.2	0.7	0.7	0.9	1.0
	succinic acid	35.2	39.2	52.3	46.0	57.9	55.5	50.2	49.4
	Threonic acid	33.6	49.1	25.2	26.7	42.0	36.6	36.0	37.7
	Azelaic acid	**93.4**	210.1*	**270.7**	35.1*	40.8	42.1	46.4	45.9
	Beta-Aminoisobutyric acid	31.1	36.2	34.7	30.2	34.0	32.1	31.1	29.5
	Quinic acid, 5-p-coumaroyl	115.1	115.2	15.0	11.7	27.1	21.7	31.1	47.4
	**Average**	**43.9**	**51.7**	**49.9**	**35.1**	**43.3**	**39.4**	**41.0**	**42.6**
**Sugars**	Lactose	83.6	95.6	146.1	131.5	128.7	127.9	**240.3**	151.6*
	Sorbitol	30.3	30.0	**130.8**	84.9*	73.5	71.3	159.9	110.2
	Mannose-6-phosphate	55.1	64.5	**101.4**	68.3*	77.4	72.7	**129.2**	93.4*
	Glucose	73.3	117.8*	**143.0**	100*	87.6	64.3	90.8	67.8
	Raffinose	36.7	47.4	24.4	27.8	9.5	11.6	**14.8**	11.1*
	Melezitose	60.5	77.8	**100.3**	66.4*	93.7	98.9	147.5	112.9
	Turanose	139.0	139.1	32.9	25.4*	24.4	17.9	22.2	17.5
	Trehalose	33.0	40.1	14.7	13.4	11.1	11.6	17.5	14.9
	Maltose	31.0	34.8	**42.1**	29.7*	28.5	33.7	43.1	40.4
	D-Xylofuranose	**40.1**	45.8*	57.0	65.1	92.2	86.2	90.2	89.1
	Fructose	**275.9**	148.4*	**211.2**	151.4*	123.5	113.7	155.4	154.5
	Rhamnose	40.0	47.0	245.1	258.6	385.2	341.9	353.4	352.1
	Sucrose	37.9	39.4	35.1	31.7	37.5	36.2	38.9	39.0
	Glycerin	29.4	32.3	30.4	29.1	29.1	28.6	29.4	29.5
	Galactitol	37.6	39.8	**116.5**	216.5*	228.4	225.6	230.9	237.4
	Erythritol	**41.3**	55.1*	87.9	104.4	79.1	69.0	97.3	102.2
	Hydroquinone-beta-D-glucopyranoside	49.7	53.9	**95.3**	77.2*	127.6	117.7	112.2	122.0
	Xylitol	33.6	38.1	**30.7**	43.4*	32.8	26.4	30.6	33.8
	Melibiose	26.7	34.5	**51.8**	37.6*	57.9	56.1	59.3	69.9
	Mannitol	57.7	68.2	163.3	151.3	279.4	258.2	285.7	348.6
	Mannose	65.1	67.3	**225.1**	174.3*	171.0	144.1	**224.8**	294.7*
	Beta-Methylglucopyranoside	39.3	41.5	**187.6**	85.9*	98.3	78.1	137.3	187.3
	**Average**	**58.7**	**60.7**	**100.1**	**87.7**	**100.4**	**92.1**	**119.2**	**118.0**
**Others**	Ethanolamine	33.5	32.9	56.2	70.3	**114.2**	96.3*	104.2	97.7
	Hydroxylamine	35.0	32.5	20.6	18.2	15.3	18.4	16.6	17.0
	Trolamine	26.3	19.9	67.6	37.1	38.3	41.3	45.9	47.3
	Glucaric acid-1,4-lactone	29.2	28.0	10.3	12.2	6.1	6.7	7.4	6.8
	Gluconic acid, 1,4-lactone	28.1	29.9	52.4	46.9	34.1	32.6	44.8	44.7
	Phosphoric acid monomethyl ester	39.8	41.1	22.4	26.4	21.0	26.7	**23.4**	33.8*
	2-Monopalmitoylglycerol	**56.7**	72.5*	**93.9**	52.6*	56.0	57.7	65.6	68.4
	Ribonic acid	30.6	31.5	27.4	26.2	20.1	22.0	23.4	23.6
	Guanosine	**53.59**	66.1*	58.9	48.8	46.3	43.3	50.9	54.4
	Uridine	48.3	62.6	**56.7**	32.8*	22.7	26.7	35.5	33.2
	Uracil	18.8	31.1	**18.6**	24.6*	26.9	24.0	**27.3**	18.5*
	Urea	29.5	31.7	30.7	15.9	18.2	18.9	21.9	23.9
	Adenosine	40.9	41.6	45.0	48.3	36.9	36.6	42.4	43.3
	Alpha-Glycerophosphorylglycerol	50.8	70.4	47.4	39.3	19.8	24.0	48.4	32.1
	Cyclohexanehexol	**27.75**	31.2*	32.3	34.8	42.0	38.8	37.0	39.1
	**Average**	**36.6**	**41.5**	**42.7**	**35.6**	**34.5**	**34.3**	**39.6**	**38.9**

Note: The values different significantly (P < 0.05) between NIL-*SS1* and Teqing were indicated with *

At the tillering stage, compared with Teqing, more metabolites increased in NIL-*SS1* (Table 2). The average levels of the AAs, OAs, sugars and SMCs in NIL-*SS1* were 75.3, 51.7, 60.7 and 41.5, respectively, which were slightly higher than those in Teqing (by 6.4%, 17.4%, 3.4% and 13.5%, respectively). Eleven of the 79 metabolites, including two AAs (tyrosine and valine), three sugars (d-xylofuranose, erythritol and glucose), three OAs (azelaic acid, pyruvic acid and butyric acid-4-hydroxy) and three SMCs (2-monopalmitoylglycerol, cyclohexanehexol and guanosine) showed significant increase in NIL-*SS1* compared with those in Teqing.

The most obvious differences were observed at the booting stage. The average levels of the AAs, OAs, sugars and SMCs in NIL-*SS1* were 92.2, 35.1, 87.7 and 35.6, respectively. Compared to those in Teqing, the AAs level was increased by 59.5%, while, OAs, sugars and SMCs were decreased by 29.7%, 12.4% and 16.6%, respectively. There were 21 metabolites identified to be significantly different. Compared with Teqing, 13 metabolites included six OAs (benzoic acid, butyric acid-4-hydroxy, caffeic acid, ferulic acid, pyruvic acid, azelaic acid) and eleven sugars (sorbitol, mannose-6-phosphate, glucose, melezitose, turanose, maltose, fructose, hydroquinone-beta-d-glucopyranoside, melibiose, mannose, beta-methylglucopyranoside) decreased significantly, and 8 metabolites included three AAs (alanine, glutamic acid, serine), two OAs (malonic acid, hexadecanoic acid), two sugars (galactitol, xylitol) and one uracil were found to be significantly increased in NIL-*SS1*.

Fewer differences were observed between two genotypes at later developmental stages (Table 2). At the flowering stage, the average levels of the AAs, OAs, sugars and SMCs in NIL-*SS1* were 77.6, 39.4, 92.1 and 34.3, respectively, which were slightly lower than those in Teqing (by 8.3%, 9.1%, 8.3% and 0.8%, respectively); At the grain filling stage, the average levels of the AAs, OAs, sugars and SMCs in NIL-*SS1* were 76.7, 42.6, 117.9 and 38.9, respectively, the level of the AAs, sugars and SMCs were slightly lower by 3.4%, 1.0%, 1.8%, respectively, while the OAs increased slightly by 4.0%, than those in Teqing.

Compared with Teqing, four metabolites (valine, glycine, caffeic acid and ethanolamine) were detected to be significantly decreased at the flowering stage, while at the grain filling stage there were nine metabolites changed significantly, including four (mannose, phosphoric acid monomethyl ester, tyrosine and pyruvic acid) that increased and five (raffinose, lactose, mannose-6-phosphate, malonic acid and uracil) decreased in NIL-*SS1*.

## Discussion

Genetic analysis indicated that *SS1* was allelic to the reported gene *NAL1* [6, 24, 25, 34]. *NAL1*, identified on chromosome 4 was reported to control the source and sink simultaneously. Meanwhile, several genes allelic to *NAL1* were identified that were not only related to plant stature, leaf *P*_*N*_, leaf size, leaf nitrogen, but also to SNP and consequently grain yield [5, 24, 34, 37]. The alleles from different varieties had different phenotype effects. For example, *SPIKE* and *GPS* are identical to *NAL1*, *SPIKE* with the allele from YTH326 induced higher total spikelet number (TSN) and FLW [24]; *GPS* with the allele from Takanari increased the *P*_*N*_, specific leaf weight and leaf nitrogen content, while *GPS* with the allele from Koshihikari has opposite effects [6]. All these results evidenced that the gene effect was influenced greatly by the genetic background [6]. In the present study, NIL-*SS1*, with an introgression of a 50.3kb segment from Lemont in the Teqing background showed significantly increased FLW, TLA, AAs content and SNP, and significantly decreased P_*N*_. Thus the function of the introgression segment with *SS1* is similar to that of *SPIKE*. Moreover, the NIL-*SS1* had higher grain yield than that of Teqing, indicating the positive effect of the introgression segment on rice yield. However, there are four genes (including *SS1*, *Os04g52440*, *Os04g52450 and Os04g52460*) on the introgression region in the NIL-*SS1*[25], whether other three genes on the introgression have phenotypic and metabolic effect on the NIL-*SS1* need further elucidated.

The physiological analysis showed that the higher yield of NIL-*SS1* might be related to the higher photosynthetic area per plant, which was reflected by the higher level of TLA. It’s known the single-leaf maximum *P*_*N*_ is partly related to the resources in the leaf which spread more thinly across a larger leaf area [38]. In the present study, the flag leaf width increased significantly while the *P*_*N*_ decreased, which was consistent with previous reports [11,39]. However, we found that NIL-*SS1* had much higher TLA than Teqing. Crop yield is believed to be more related to the total photosynthesis of the whole canopy, rather than the photosynthesis of a particular leaf. It’s reported, the higher yield of modern crop cultivars has been achieved mainly by increasing the light-intercepting area of leaves [10,12]. Thus, the higher yield of the NIL-*SS1* could partly attribute to the higher TLA.

The metabolic profile analysis revealed that the influence of the introgression segment on metabolism was highly dependent on the developmental situation, and the most significant influence occurred at the booting stage, followed by tillering stage (Figs 1 and 2). The booting stage is the critical chronological stage to determine the spikelet number, and is consequently the critical period for yield determination [40–42]. From the results, we observed that compared with that in Teqing, the metabolites phenotype of NIL-*SS1* at the booting stage was more closely associated with that at the flowering stage (Fig 2); moreover, the levels of most sugars, especially those involved in the degradation of the sucrose and starch and the metabolites related to the second metabolism were lower, while some OAs in TCA and the AAs were significantly enriched in the leaves of NIL-*SS1* (Fig 3). The heterotrophic organ systems in plants are dependent upon amino acid and sugar import for normal growth and development. For example, as much as 80% of the carbon assimilated during photosynthesis is exported from the leaf to satisfy the metabolic needs of the non-photosynthetic cells [43]. The import of photosynthates is regulated by the metabolic activities associated with cell division and/or cell enlargement within the sink [12]. At the booting stage, the organs for spike differentiation are an active sink that has a strong, competitive ability to absorb more assimilates [44], which is an energy-dependent process [12]. Thus, we concluded that the more active accumulation of metabolites, especially of the sugars and OAs, in young growing spikes was the possible explanation for the higher SNP in NIL-*SS1*. Furthermore, the significantly higher AAs contents were consistent with result for *NAL1*, which controls the leaf nitrogen content [6]. Nitrogen is the macronutrient required in the highest amounts by plants [45,46], and the AAs serve as important nitrogen carriers in plants [45,47–48], which are the first stable products of inorganic N assimilation [47] and are the building blocks for proteins. Thus, in the present study, the higher amino acids levels in the leaves of NIL-*SS1* at the booting stage would be beneficial for its fast growth and young spike growth.

At the tillering stage, compared with Teqing, the levels of most of the minor sugars, nucleotises, the metabolites involved in the degradation of sucrose, starch and cell wall, related to the redox reaction were higher in NIL-*SS1*. The photosynthates were consumed mainly for plant growth and biomass accumulation at the vegetative stage. The higher metabolites levels in the leaves of NIL-*SS1* were possibly related to its faster growth rate and larger leaf size.

Two metabolites, azelaic acid and butyric acid-4-hydroxy, varied greatly between NIL-*SS1* and Teqing across the tillering and booting stages, while gluconic acid-6-phosphate and threonic acid, and caffeic acid and beta-methylglucopyranoside changed obviously at the tillering and booting stages, respectively. The significant differential change in the azelaic acid and butyric acid-4-hydroxy levels at the two stages indicated their possible active functions in NIL-*SS1*’s special performance. Azelaic acid primes systemic defenses in Arabidopsis [49]. When applied locally at certain concentrations, azelaic acid promoted disease resistance in the distal organs [50]. Butyric acid-4-hydroxy is the precursor of the succinic acid, which is one of the key components in the tricarboxylic acid cycle (TCA). The potential relationship between *SS1* target metabolites and leaf size will be analyzed in the future.

## Conclusions

Introgression line NIL-*SS1*, carrying the Lemont derived segment (50.3kb) which harbored *SS1* gene within the Teqing (*indica*) genetic background, was developed by marker-assisted selection. NIL-*SS1* had broader leaves and higher grain yield, with more spikelet number per panicle, than Teqing. To understand the influence of the introgression segment on plant growth, physiological and morphological traits, and grain yield, were investigated. Moreover, metabolic profiling analyses were conducted using GC-MS technology to uncover the changes in metabolites patterns in NIL-*SS1*. The results showed that NIL-*SS1* has higher levels in flag leaf width, total leaf area, spikelet number per panicle, and lower levels in photosynthetic rate; significant metabolic differences were observed across different stages, with greatest differences between NIL-*SS1* and Teqing occurring at the booting stage. The introgression segment induced more active competition for sugars and organic acids from leaves to young spike growth point, which resulted in a higher spikelet number per panicle. The contents of azelaic acid and butyric acid-4-hydroxy varied significantly between two genotypes at the tillering and booting stages. The results indicated that introgression segment harbored with gene *SS1* could improve the plant growth rate, which was beneficial to photosynthate accumulation during active growth point.

## Supporting Information

S1 Table ANOVA results for 79 metabolites in leaves of two rice genotypes, Teqing and NIL-*SS1*, measured at four different growth stages (tillering, booting, flowering and grain filling stages). (XLSX)

S2 Table The contributions of each of the 79 metabolites to the first three principal components (PC). (XLSX)
